# Exhaled breath particles as a novel tool to study lipid composition of epithelial lining fluid from the distal lung

**DOI:** 10.1186/s12890-023-02718-8

**Published:** 2023-11-03

**Authors:** Per Larsson, Olaf Holz, Grielof Koster, Anthony Postle, Anna-Carin Olin, Jens M. Hohlfeld

**Affiliations:** 1 Occupational and Environmental Medicine, School of Public Health and Community Medicine, Institute of Medicine, Sahlgrenska Academy, University of Gothenburg, Gothenburg, Sweden; 2 Department of Clinical Airway Research, Fraunhofer Institute for Toxicology and Experimental Medicine, 30625 Hannover, Germany; 3 German Center for Lung Research, Biomedical Research in Endstage and Obstructive Lung Disease Hannover (BREATH), Hannover, Germany; 4 Faculty of Medicine, University of Southampton, Southampton, UK; 5 Hannover Medical School, Department of Respiratory Medicine, Hannover, Germany

**Keywords:** Airway inflammation, Endotoxin challenge, LPS, Human experimental exposure

## Abstract

**Background:**

Surfactant phospholipid (PL) composition plays an important role in lung diseases. We compared the PL composition of non-invasively collected exhaled breath particles (PEx) with bronchoalveolar lavage (BAL) and induced sputum (ISP) at baseline and following endotoxin (LPS) challenges.

**Methods:**

PEx and BAL were collected from ten healthy nonsmoking participants before and after segmental LPS challenge. Four weeks later, PEx and ISP were sampled in the week before and after a whole lung LPS inhalation challenge. PL composition was analysed using mass spectrometry.

**Results:**

The overall PL composition of BAL, ISP and PEx was similar, with PC(32:0) and PC(34:1) representing the largest fractions in all three sample types (baseline PC(32:0) geometric mean mol%: 52.1, 56.9, and 51.7, PC(34:1) mol%: 11.7, 11.9 and 11.4, respectively). Despite this similarity, PEx PL composition was more closely related to BAL than to ISP. For most lipids comparable inter-individual differences in BAL, ISP, and PEx were found. PL composition of PEx was repeatable. The most pronounced increase following segmental LPS challenge was detected for SM(d34:1) in BAL (0.24 to 0.52 mol%) and following inhalation LPS challenge in ISP (0.45 to 0.68 mol%). An increase of SM(d34:1) following segmental LPS challenge was also detectable in PEx (0.099 to 0.103 mol%). The inhalation challenge did not change PL composition of PEx.

**Conclusion:**

Our data supports the peripheral origin of PEx. The lack of PL changes in PEx after inhalation challenge might to be due to the overall weaker response of inhaled LPS which primarily affects the larger airways. Compared with BAL, which always contains lining fluid from both peripheral lung and central airways, PEx analysis might add value as a selective and non-invasive method to investigate peripheral airway PL composition.

**Trial registration:**

NCT03044327, first posted 07/02/2017.

**Supplementary Information:**

The online version contains supplementary material available at 10.1186/s12890-023-02718-8.

## Background

Alterations of surfactant phospholipid (PL) composition play an important role in acute and chronic lung diseases [1–4]. Contributors to changes of PL composition in epithelial lining fluid (ELF) include alterations in pulmonary surfactant synthesis, catabolism, and re-uptake in alveolar type II cells, infiltration of plasma lipoproteins into the airways, contamination with cell membrane fragments from necrotic cells, and the release of micro-particles from inflammatory cells. The PL composition of ELF recovered from healthy lungs by bronchoalveolar lavage (BAL) is dominated by lung surfactant [5]. However, the extent of disease-related changes to the PL composition of ELF is not clear, as BAL samples both alveolar and airway regions of the lung (distal from the bronchoscope position in the 3^rd^- 4^th^ generation, ~5 mm bronchial diameter).

There is currently a broad consent that exhaled breath particles (PEx) are formed when collapsed distal airways (~14^th^-17^th^ generation, ~0.5 mm diameter) re-open during inhalation [6–8]. Therefore, PEx are thought to represent pure ELF from the very peripheral lung [9] without substantial contamination of secretions from larger airways. There are different methods to collect particles from breath. Exhaling into cooled condenser devices is commonly used. The collection of this exhaled breath condensate (EBC) is simple and does not require expensive devices, but it suffers from a large and unknown aqueous dilution leading to limitations regarding reproducibility [10]. In contrast, the collection of exhaled breath particles by impaction is a more efficient way to collect undiluted ELF from breath [11, 12, 8, 13]. In addition, particle counting by size during the sampling procedure allows determination of the mass of collected PEx, which is of utmost importance as PEx emission shows a large inter-individual variation [8, 13]. It also allows collection of a predetermined mass of collected PEx that is suitable for the intended biochemical analysis.

So far, nothing is known about the PL composition of PEx at baseline and under inflammatory conditions. Therefore, we collected PEx in an experimental endotoxin challenge and compared its PL composition with BAL and ISP samples. In this two-period study, healthy volunteers underwent a segmental LPS challenge with bronchoscopies and BAL in the first part and a whole lung inhalation LPS challenge with sputum induction in the second part resulting in a clear inflammatory response in BAL and ISP [14]. The inflammatory signal correlated with gas transfer using hyperpolarized ^129^Xe MRI [15], and we detected increases in some breath aldehyde levels [16]. Interestingly, the LPS-driven inflammatory response was mirrored by increased levels of IL-6 and IL-8 in PEx samples [17]. Here we report data on PL composition from PEx samples of this study with the aim to compare the PL composition with BAL and ISP samples and to evaluate if LPS induced PL changes could also be detected in PEx.

## Methods

### Study design

We included ten healthy non-smoking participants (7 male and 3 females, smoking history < 1 pack-year, mean (SD) age 38 ± 10y), with normal lung function (FEV_1_ 101 ± 14% pred.; FEV_1_/FVC 76 ± 3%) for PEx sampling twice before, and twice after (5, 21 h) a segmental endotoxin (lipopolysaccharide, LPS) challenge. Following a four-week washout period, PEx were also collected during the week before and 5 h after a whole lung LPS inhalation challenge. For a schematic study outline refer to [17]. PEx lipid composition was compared to BAL which was collected prior to and 24 h following segmental LPS challenge and to ISP collected 6 h following LPS inhalation challenge. The study was conducted in accordance with local laws, regulations and GCP guidelines (CPMP/ICH/135/95), the protocol was approved by the ethics committee of the Hannover Medical School and registered at Clinicaltrials.gov (07/02/2017, NCT03044327).

### Sample collection

PEx were collected by impaction on polytetrafluoroethylene (PTFE) membranes (FHLC 02500, Merck, Germany) using the PExA® instrument (PExA AB, Sweden). Subjects were asked to perform deep breathing maneuvers to increase the PEx emission [11, 8]. Between breathing maneuvers, where PEx collection is made, participants could recover with relaxed breathing of particle free air. We aimed to collect a total particle mass of 240 ng, and this amount was achieved by most subjects. Half of the collected material was used for the lipid analysis in this study. A list of the individual amounts collected at each visit can be found in the supplement of [17]. The PEx mass was chosen based on the amount needed for the chemical analysis. The segmental LPS challenge and the bronchoscopy procedure with collection and cellular analysis of BAL have been reported in detail before [18]. The inhalation challenge was performed with a low dose of LPS (2 µg) and efficient deposition as described in detail in [19]. The LPS solution was nebulized using an Aeroneb solo nebulizer (Inspiration Medical, Bochum, Germany), with an MMAD of 3.1 µm. The particle size and the specific standardized breathing maneuver used, are considered to result in a predominantly central airway deposition [20]. The induction, processing and analysis of ISP was performed according to standard procedures [14].

### Lipid analysis

PEx samples were extracted from the PTFE membrane using [methanol:dichloromethane:40 mM ammonium acetate(aq)] in the ratios [6:3:2], which was also used as diluent for lipids extracted from BAL and ISP. The latter were extracted using Bligh and Dyer two-phased extraction. The organic phase with lipids was recovered, evaporated under nitrogen, then solubilized in 100 µl of methanol and finally diluted to the optimal assay concentration before analysis by reverse-phase liquid chromatography – mass spectrometry (LC–MS). For further details please refer to the online supplement.

### Statistics

Relative quantification was made by expressing the molar amount of each individual lipid as a percentage of the sum of lipids analyzed in the sample. All samples were analyzed in duplicate injections and the average was used. Relative standard deviation for duplicates had an average of 2.4% (range 0–14%) and QC samples were distributed throughout the run to assess method stability during the run. Data was log transformed and was provided as geometric mean and standard deviation. Repeated measures ANOVA was used for the comparison between visits. Multivariate analysis was made using the SIMCA software (SIMCA, Sartorius AG, Germany). Difference between sampling method were evaluated by Orthogonal Partial Least Squares Discriminant Analysis (OPLS-DA). The OPLS-DA model cross validation was based on a grouping by subject id, so that all data from one subject was removed from the model and then predicted by the model. The coefficients of variation (CV) for repeated PEx measurements were calculated separately for each lipid. For this, the standard deviation of all subjects was divided by the mean value for all subjects. As the CVs were not normally distributed, we provided their medians and IQR for all lipids in the results. A value below 10 can be considered as good. No correction for multiple testing was done due to the exploratory nature of our study.

## Results

Similar to BAL, the PL composition of PEx and ISP was dominated by lung surfactant phospholipids, with PC(32:0) being the most abundant of the targeted lipids. Table 1 shows PL composition by molecular species normalized to the total amount of lipids (mol%). Relative concentrations are directly comparable between PEx, BAL and ISP, and show the similarity in the PL composition. This is not possible with molar concentrations, which are provided in the online supplement (Table S1).

**Table 1:** Data of fourteen lipids in BAL, PEx and ISP (mol%) before and after segmental and inhalation LPS challenge

	**Segmental challenge**							**Inhalation challenge**			
	**BAL** **mol** **%**			**PEx** **mol** **%**				**PEx** **mol** **%**		**ISP** **mol** **%**	
**visit**	**V3**	**V4**	**V4**	**V1**	**V2**	**V3**	**V4**	**V5**	**V6**	**V5**	**V6**
**lipid**	**Baseline**	**Saline**	**24 h post LPS**	**Baseline**	**Baseline**	**3h post LPS**	**21h post LPS**	**Baseline**	**5h post LPS**	**Baseline**	**6h post LPS**
**PC(30:0)**	9.72(1.21)	9.02(1.12)	8.30(1.14)↓***	10.35(1.13)	9.61(1.27)	10.43(1.21)	9.81(1.16)	9.56(1.17)	9.68(1.21)	7.67(1.15)	8.08(1.20)
**PC(31:0)**	2.17(1.17)	2.18(1.13)	2.14(1.12)	2.08(1.20)	1.95(1.30)	2.22(1.17)	2.13(1.14)	2.04(1.18)	2.01(1.25)	2.02(1.12)	2.01(1.15)
**PC(32:0)**	52.12(1.06)	53.01(1.04)	50.73(1.05)↓**	51.72(1.04)	51.85(1.05)	51.01(1.05)	50.89(1.04)	51.40(1.04)	51.53(1.03)	56.87(1.03)	55.41(1.04)↓*
**PC(32:1)**	8.77(1.13)	9.05(1.15)	8.90(1.14)	9.33(1.11)	9.26(1.13)	9.51(1.17)	9.94(1.14)	9.98(1.16)	9.59(1.18)	7.48(1.19)	7.38(1.25)
**PC(33:0)**	1.67(1.16)	1.73(1.13)	1.66(1.09)	1.52(1.16)	1.51(1.18)	1.70(1.15)	1.63(1.14) →*	1.54(1.13)	1.52(1.14)	1.77(1.14)	1.66(1.09)
**PC(34:0)**	2.02(1.16)	2.03(1.16)	1.80(1.15)↓***	2.09(1.20)	2.17(1.21)	2.08(1.20)	2.00(1.17)	2.02(1.14)	2.06(1.18)	2.20(1.16)	2.17(1.16)
**PC(34:1)**	11.74(1.06)	11.94(1.07)	12.63(1.07) ↑***	11.35(1.13)	12.16(1.08)	11.94(1.06)	12.32(1.07)	12.18(1.06)	11.95(1.09)	11.93(1.06)	11.83(1.10)
**PC(34:2)**	5.25(1.15)	5.04(1.14)	6.36(1.14)↑***	5.78(1.14)	5.39(1.12)	5.33(1.14)	5.43(1.11)	5.46(1.17)	5.67(1.15)	4.23(1.19)	4.66(1.16)
**PC(36:1)**	1.05(1.19)	1.02(1.20)	1.14(1.21)↑***	0.654(1.24)	0.716(1.17)	0.712(1.16)	0.670(1.18)	0.67(1.13)	0.66(1.23)	1.14(1.14)	1.24(1.28)
**PC(36:2)**	2.62(1.22)	2.47(1.19)	3.05(1.18)↑***	2.43(1.16)	2.56(1.15)	2.44(1.21)	2.60(1.16)	2.56(1.14)	2.53(1.18)	2.23(1.19)	2.45(1.19)↑**
**PC(36:3)**	1.56(1.22)	1.46(1.23)	1.71(1.18)↑**	1.64(1.23)	1.58(1.20)	1.50(1.24)	1.57(1.21)	1.57(1.23)	1.63(1.23)	1.09(1.30)	1.22(1.22)
**PC(** **O-32:0)**	0.25(1.26)	0.24(1.31)	0.31(1.34)↑***	0.274(1.26)	0.241(1.24)	0.244(1.29)	0.248(1.27)	0.24(1.24)	0.24(1.28)	0.29(1.25)	0.39(1.46)↑**
**PC(O-32:1)**	0.28(1.19)	0.28(1.26)	0.31(1.27)↑**	0.256(1.20)	0.250(1.19)	0.231(1.29)	0.255(1.26)	0.25(1.18)	0.25(1.24)	0.21(1.24)	0.20(1.19)
**SM d34:1**	0.24(1.29)	0.17(1.26)	0.52(1.44)↑***	0.099(1.19)	0.100(1.19)	0.092(1.17)	0.103(1.21)↑*	0.096(1.20)	0.108(1.25)~	0.45(1.28)	0.68(1.59)↑**

Geometric means and standard deviations (as factor)

*V* visit, *BAL* bronchoalveolar lavage, *PEx* exhaled breath particles, *ISP* induced sputum, *LPS* endotoxin, ***=*p*<0.001, **=*p*<0.01, *=*p*<0.05~=*p*=0.08

Repeated measures ANOVA for the three BAL samples and for the PEx samples (V2, V3,V4) after segmental challenge

Pairwise t-test for PEx and induced sputum samples for the inhalation challenge. The arrow indicates the direction of change in molar ratios

**Fig. 1:**
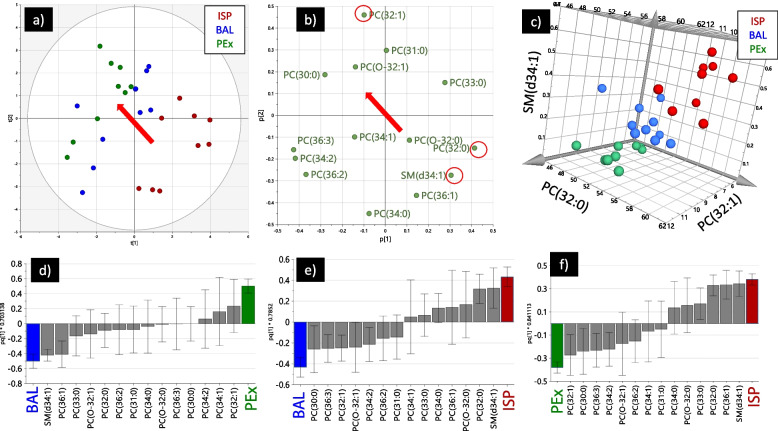
**a** Principal component score plot with induced sputum (ISP) in red, bronchoalveolar lavage (BAL) in blue and exhaled particles (PEx) in green. Principal component analysis (PCA) model with two components. Unit variance scaling was used. **b** PCA loading plot that illustrates which lipids were associated to the principal components in **a**. Direction of sample type separation indicated by red arrow. Three of the most influential lipids separating the sample types are highlighted with red circles. **c** Plot of the mol% of the three lipids highlighted in **b**. **d** Orthogonal partial least square analysis (OPLS-DA) for pairwise comparison of BAL and PEx to identify difference for each lipid with a 95% confidence interval. The model parameters and model validation are presented in the supplement. **e** OPLS-DA difference between BAL and ISP. **f** OPLS-DA difference between PEx and ISP

PL composition of PEx, BAL and ISP at baseline conditions (BAL from visit 3 prior to segmental LPS challenge, PEx and sputum from visit 5 prior to inhaled LPS challenge) was compared by multivariate analysis. From the unsupervised principal component analysis (PCA) model, a spatial grouping of PEx, BAL and ISP could be observed in the score plot (Fig. 1a). The loading plot (Fig. 1b) shows how influential each lipid was for this grouping. Using only three of the identified lipids, which are highlighted as important in then loading plot (Fig. 1b), it was possible to fully separate sample types into discrete groups based on their mol% (Fig. 1c).

Supervised OPLS-DA is a more powerful method to summarize the lipid variance associated to the sampling methods than the unsupervised PCA method that summarize the total lipid variance in the dataset. The most influential lipids for sample type discrimination in the OPLS-DA model (Fig. 1d-f) were similar to the ones identified in the PCA analysis, indicative of a robust OPLS-DA model. The importance of a lipid for defining the class can be interpreted from the loading value (bar) divided by the width of the 95% confidence interval (whiskers). The respective S-plots are provided in the online supplement. As indicated in Fig. 1d-f the difference in lipid composition between PEx and ISP was much larger than the difference between PEx and BAL, because 10/14 lipids were significantly different compared to just 3/14 lipids when comparing PEx and BAL. This data was confirmed by a mixed model analysis which showed that 12/14 lipids were different between PEx and ISP, while only 3/14 lipids showed differences between PEx and BAL (summary table in the online supplement).

**Fig. 2:**
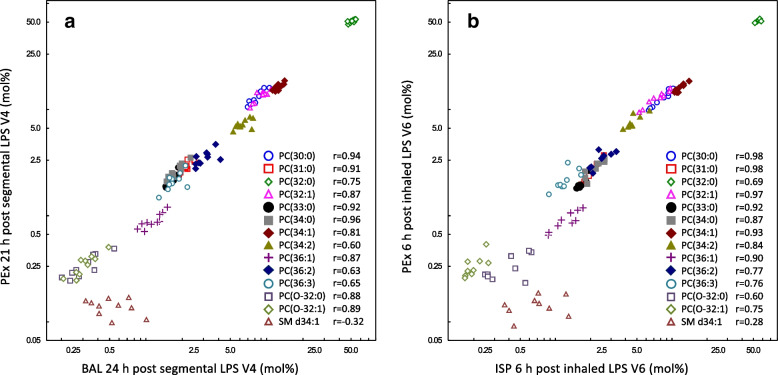
**a** Correlation between BAL and PEx for all lipids. **b** Correlation between ISP and PEx for all lipids. The respective correlation coefficients for each lipid are provided within each figure

Inter-individual differences in lipid levels between subjects were observed as previously reported [20]. Moreover, differences in PC lipids correlated well between PEx and BAL (Fig. 2a), as well as between PEx and ISP samples (Fig. 2b). The comparisons between PEx samples taken at different time points are shown in the online supplement (Figure S1). The closest relationship for the inter-individual differences in the mol% was observed between PEx samples collected at 3 h (visit 3) and 21 h (visit 4) after segmental LPS, followed by the samples taken 6 days apart at the first (visit 1) and second baseline visits (visit 2). For samples taken more than 4 weeks apart (visit 2 vs. visit 5) the relationship was weaker (Figure S1). We computed the respective coefficients of variation (CV). For the visit 3 vs. visit 4 the median CV (IQR) value was 3.4 (5.2), for visit 1 vs. visit 2 it was 4.8 (8.0) and for visit 2 vs. visit 5 it was 7.5 (8.2). For the shortest time period between PEx collections (18 h) we also assessed the repeatability. The Bland–Altman plots as well as the ICCs, which are all > 0.84, except for three lipids, are provided in the online supplement (Figure S2).

Both segmental and inhalation LPS challenges induced an inflammatory response characterized by a significant influx of neutrophils [14] and an increase in inflammatory mediators [17]. The most pronounced PL changes were observed in BAL of the segment, which was challenged with LPS (Table 1). Most lipids showed significant changes compared to the control segments, especially we saw an increase in unsaturated lipids. PEx samples, which originated and were collected from all five lobes of the lung, and not just the challenged segment, showed only small differences compared to the respective baseline sample. A small but significant increase could be observed for the level of SM(d34:1) in PEx, which also showed the largest response to LPS in the challenged segment. Following inhalation challenge, PL in ISP substantially reflected the previous BAL changes (Table 1), but to a much smaller extent. Importantly, there were again only minimal changes in PEx following inhalation challenge.

## Discussion

Our data demonstrates that, despite an overall similarity, the PL composition of PEx is more closely related to BAL than to ISP, which is in line with the proposed peripheral origin of PEx. PEx analysis was capable to show the inter-individual differences in the level of some lipids that were observed in BAL and ISP. Repetitive PEx sampling demonstrated that PL analysis in PEx yielded very reproducible data. LPS affected most targeted lipids in the challenged lobe but only the increase of SM(d34:1) could be detected in PEx, which are sampled from all lobes. The inhalation challenge showed a similar, but an overall weaker response. In addition, it most likely acted predominantly in the central airways and was therefore not detectable in PEx. The lipid composition of PEx provide further circumstantial support for the suggested mechanism for particle formation during re-opening of small peripheral airways. The peripheral lung origin of PEx could be an advantage for the study of PL composition compared to BAL, which naturally also contains portions of larger airways.

We standardized our LC/MS analysis of lipids, by always using the same amount of BAL, ISP and PEx collection filters extraction fluid. Internal standard was added to these samples prior to the processing, however, this cannot account for variable PL recovery and dilution during collection of BAL and ISP or potential dilution effects due to LPS-induced leakage of fluid into the airways. Therefore, normalization of lipid data was performed. There are two major approaches. First, reference to a major or abundant lipid, e.g. PC(32:0) can be made with computation of the ratio of each lipid relative to this reference. This bears the risk, that the selected reference lipid itself is affected by the LPS challenge (or disease). Therefore, we normalized each lipid to the sum of all analysed lipids. The resulting mol% have the additional benefit, that the relative levels between BAL, ISP and PEx can be better compared. Due to the lack of normalization not all changes shown in Table S1 are comparable to those shown in Table 1.

The OPLS-DA analysis was performed to identify differences in the lipid profile between sampling methods. For this analysis we selected the BAL baseline visit, and the ISP and PEx baseline visits which were performed on the same day. There is no visit, for which baseline visits from all three methods are available and we are aware that this approach favors a closer similarity between PEx and ISP Nevertheless, our analysis showed that there was a greater similarity between BAL samples and PEx samples. With induced sputum being derived from the more central airways [21] and with BAL sampling both the proximal and distal lung, our data supports the commonly accepted distal lung origin of PEx. However, despite multiple indirect evidence, we acknowledge that there is still no final proof that PEx are generated by the reopening of collapsed very small airways.

PC(32:0) or dipalmitoyl-phosphatidylcholine (DPPC) is an essential PL for maintaining low surface tension of airway lining fluid. It displayed the lowest inter-individual variability, indicating that its function requires a well-defined concentration. It is known that inter-individual differences between lipid levels exist [22] and we observed that such differences correlated between BAL and PEx, but also, to a lesser extent, between ISP and PEx. Inter-individual differences could also be observed between sequentially collected PEx samples, especially between samples collected within short time intervals, suggesting that the changes in lipid levels over of longer periods of time represent more biological and not methodological variation. The overall levels of the variation coefficients were below 10% for all comparisons indicating a high level of precision for repeated measurements in PEx.

Both segmental and inhalation LPS challenge resulted in a clearly detectable neutrophilic influx [14] and in an increase in inflammatory cytokines [17]. In the LPS challenged segment, we also detected a small, but statistically significant change in the level of lipids. Inflammation increased the BAL concentration of unsaturated and reduced saturated lipids, in line with Heeley et al*.* [23]. The largest LPS-induced change was observed for SM(d34:1), which is a minor component of lung surfactant, but present in much higher concentration in both cell membranes and blood plasma [24, 25]. Therefore, it is speculated that the change in SM(d34:1) is more driven by cellular inflammation in the distal lung than by altered surfactant synthesis.

Overall, the changes in PL composition in PEx were small. It could be argued, that PEx analysis is therefore not sensitive enough to detect LPS induced inflammatory changes. However, it needs to be kept in mind, that the segmental LPS challenge is limited to one lobe of the lung, while PEx are sampled from the small airways of all five lobes.

The inhalation LPS challenge elicited only minimal PL changes in PEx and a smaller, but basically similar inflammatory response in ISP as compared to BAL from the challenged segment. It could be argued again, that PEx analysis was not sensitive enough to detect these small LPS induced changes. But it needs to be considered, that based on the inhalation maneuver and particle size of the inhaled LPS, the majority of LPS-induced changes in PL composition are probably limited to the larger airways. The small increments in PEx SM(d34:1) would then indicate that inflammation-related changes to proximal lung PL composition have to be substantial before they can be detected in samples from the distal lung. Based on our data it is tempting to conclude that the PExA analysis could even be a better tool than BAL to investigate PL composition in the peripheral lung. Not only because it is non-invasive, which will enable new study designs with more frequent sampling, but also because it samples ELF undiluted and with potentially less contaminations from larger airways as compared to BAL.

### Limitations

Due to the complex design and long duration of the study we were only aiming to include 10 participants. Naturally we used BAL to analyse the effects of segmental LPS challenge. Due to ethical reasons, we restricted the analysis of the inhalation challenge to ISP, which due to its more central origin is not directly comparable to BAL. Therefore, our data interpretation remains speculative in some aspects and generates hypothesis which would need to be proven in different study designs. The LPS induced changes were not large in the healthy subjects. This might have been different in asthma or COPD patients, however, due to the variability in disease status between patients and over time, such a study would have required a much larger group of subjects.

## Conclusion

Taken together, this study provides evidence that the PL composition of PEx is more closely related to BAL than to ISP, and that PEx analysis is robust and apparently especially suited to investigate the PL composition in the distal lung.

### Supplementary Information

**Additional file 1. **

## Data Availability

The datasets generated and/or analysed during the current study are not publicly available due data protection regulations but are available from the corresponding author on reasonable request.

## Abbreviations

BAL: Bronchoalveolar lavage
CV: Coefficients of variation
EBC: Exhaled breath condensate
ELF: Epithelial lining fluid
FEV1: Forced expiration in in the first second
FEV1/FVC: Tiffenau-Index (forced expiration in the first second relative to forced vital capacity)
IQR: Interquartile range
ISP: Induced sputum
LC–MS: Liquid chromatography Mass spectrometry
LPS: Endotoxin
MMAD: Median mass aerodynamic diameter
OPLS-DA: Orthogonal partial least squares discriminant analysis
PCA: Principal component analysis
PEx: Exhaled breath particles
PL: Surfactant phospholipid
PTFE: Polytetrafluoroethylene
SD: Standard deviation
