# Supplementary material for: Exhaled breath particles as a novel tool to study lipid composition of epithelial lining fluid from the distal lung
Source: BMC Pulm Med. 2023 Nov 3;23:423. doi: 10.1186/s12890-023-02718-8 (PMC10623716; doi:10.1186/s12890-023-02718-8)
Supplement: Supplementary file 1 — Additional file 1. [file 12890_2023_2718_MOESM1_ESM.docx]

**Online Supplement**

**Exhaled breath particles as a novel tool to study lipid composition of epithelial lining fluid from the distal lung**

P Larsson^*,1^, O Holz^*,2,3^, G Koster^4^, A Postle^4^, AC Olin^1^, JM Hohlfeld^2,3,5^

1 Occupational and Environmental Medicine, School of Public Health and Community Medicine, Institute of Medicine, Sahlgrenska Academy, University of Gothenburg, Gothenburg, Sweden.

2 Fraunhofer ITEM, Clinical Airway Research, Hannover, Germany.

3 German Center for Lung Research, Biomedical Research in Endstage and Obstructive Lung Disease Hannover (BREATH), Hannover, Germany.

4 Faculty of Medicine, University of Southampton, UK.

5 Hannover Medical School, Department of Respiratory Medicine, Hannover, Germany.

*contributed equally.

Corresponding author:

Dr. Olaf Holz

Department of Clinical Airway Research,

Fraunhofer Institute for Toxicology and Experimental Medicine,

30625 Hannover, Germany

Phone: +49-511-5350-8141

Fax: +49-511-5350-8250

olaf.holz@item.fraunhofer.de

LC-MS method details:

For the reverse-phase chromatography, water was used as weak eluent and methanol as the strong eluent. Both mobile phases had 1% (v/v) formic acid and 0.2% (v/v) ammonia added as modifiers. Separation was carried out using a gradient elution on a Waters ACQUITY BEH C8 Column (no. 186002878). A set of 14 lipids were detected on a Waters Xevo triple quadrupole mass spectrometer using targeted multiple reaction monitoring for the phosphatidylcholine (PC) head group transition that is typical for PC and sphingomyelin (SM) lipids, [M+H]+>184.1 m/z. Molar quantification was made using PC(14:0/14:0) as an internal standard. The lipid PC(14:0/14:0) was not found at a significant level in any of the sample types before spiking. The target amount of spiked internal standard (IS) was to obtain a signal that was around 50% of the major lipid PC(32:0). It has been shown that the [M+H]+>184.1 m/z transition gives similar response factor for the most common PC lipids even if response factor decline somewhat with increasing molecular mass [1], therefore one IS can provide reasonably good accuracy for quantification.

**Reference**

**1** Brügger B, Erben G, Sandhoff R, Wieland FT, Lehmann WD. Quantitative analysis of biological membrane lipids at the low picomole level by nano-electrospray ionization tandem mass spectrometry. *Proceedings of the National Academy of Sciences of the United States of America* 1997; 94: 2339–2344.

**Table S1.** Mol amount per sample in BAL (100µL), ISP (100µL) and PEx (120ng)


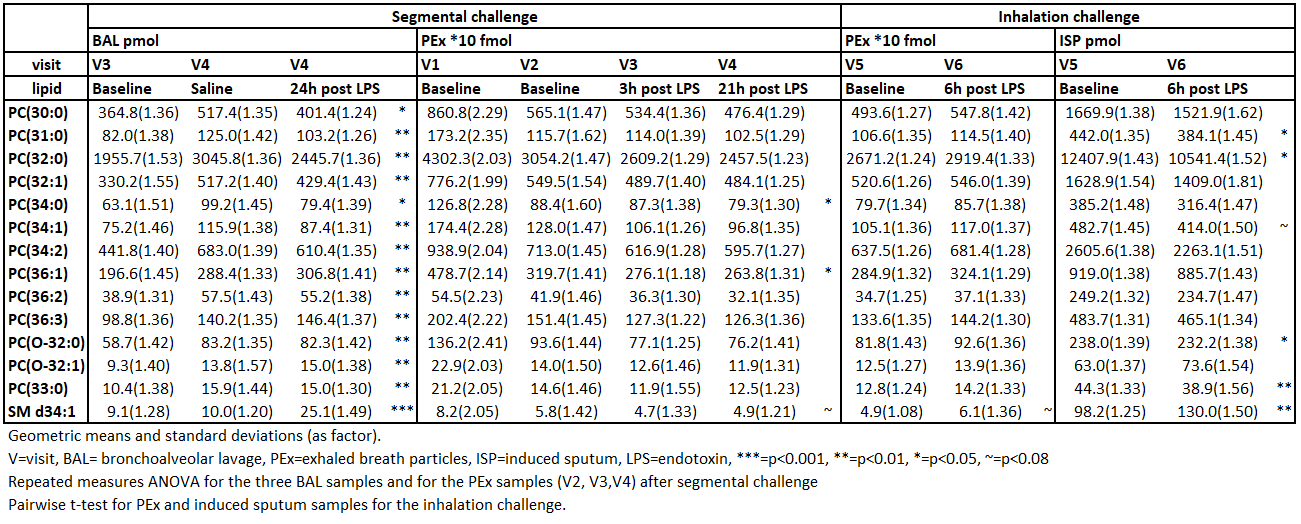


Please note that absolute amount of lipids highly depend on the variable amount of retrieved material and therefore some changes shown in table S1 deviate from the changes shown in the normalized mol % data of table 1. However, the data in table S1 shows the consistency within each matrix with respect to the absolute sampled amount of lipids.

**Figure S1.** Comparison of PEx data (a-c) and BAL data (d) repeated at different time points. **a:** PEx 18 h apart between visit 3 and visit 4. **b:** PEx 6 days apart between visit 1 and visit 2. **c:** PEx >28 days apart between visit 2 and visit 5. **d:** BAL 24 h apart between visit 3 and visit 4 for baseline BAL sample from control segment and BAL sample from saline challenged segment.


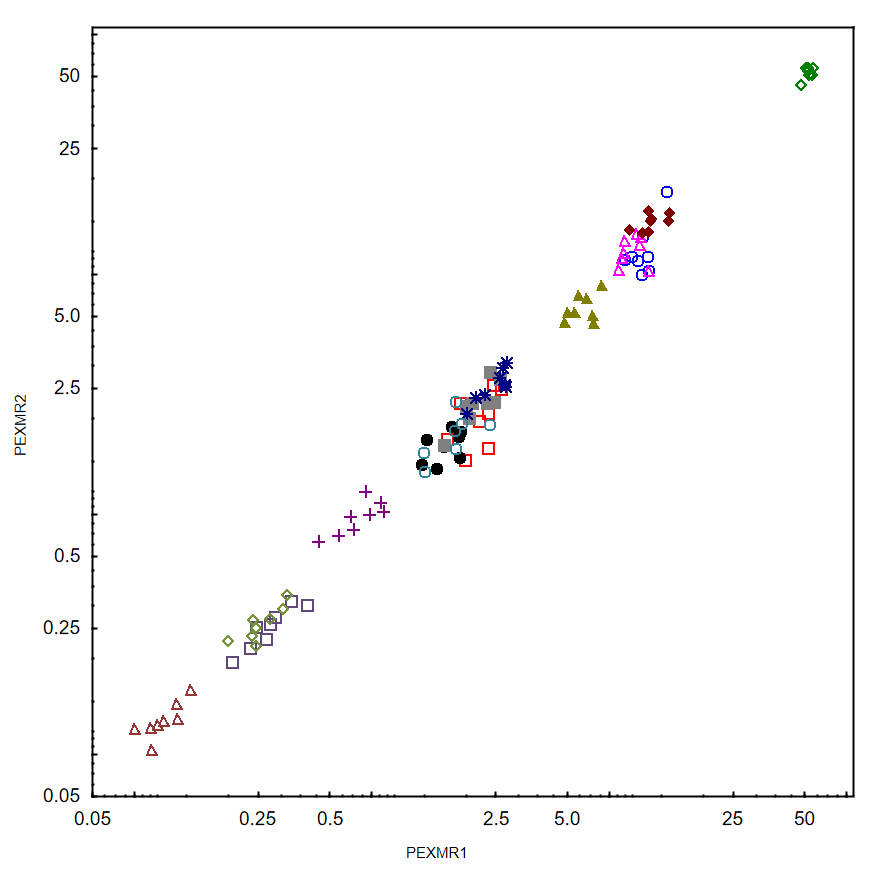

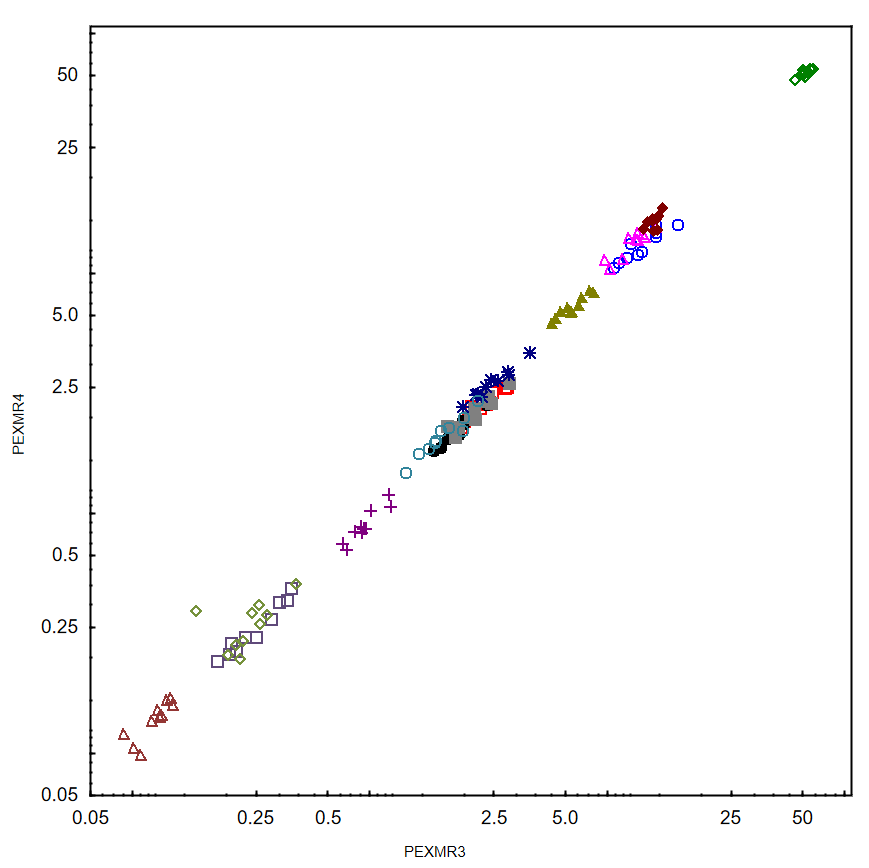


**b**

**a**

PEx 21h post segmental LPS V4


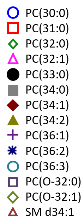

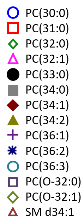


PEx baseline V2


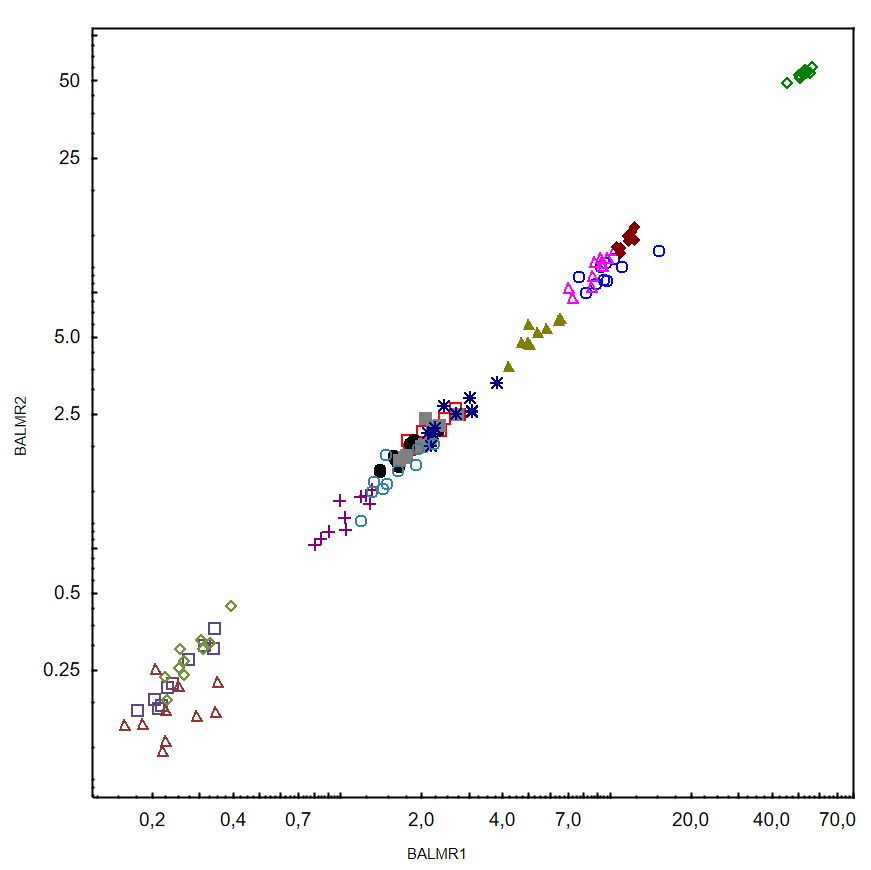

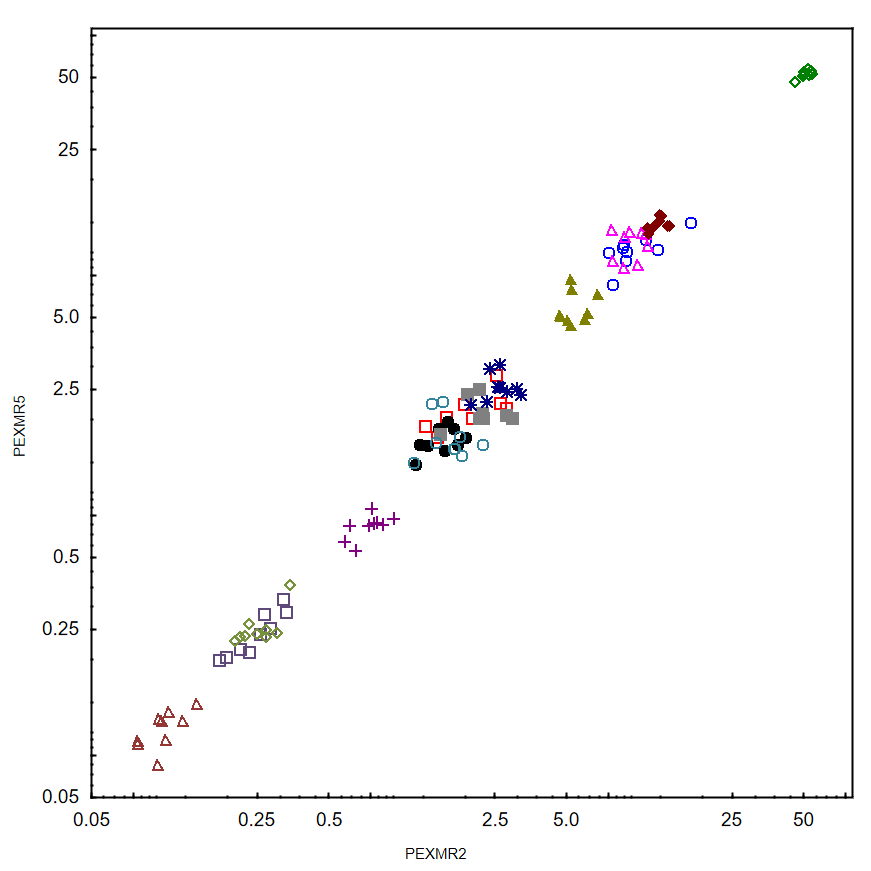


PEx baseline V1

PEx 3h post segmental LPS V4

**d**

**c**

BAL saline challenge segment V4


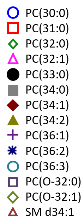

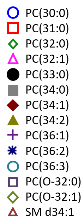


PEx baseline V5

BAL baseline V3

PEx baseline V2

**Figure S2.** Repeatability of PEx analysis. Comparison between visit 3 and visit 4 (18 h time period) for all lipids. Bland Altman plots showing mean vs difference with 2 standard deviations as limits. The table lists the respective intraclass correlation coefficients (ICC) were derived from one-way ANOVA tables as the ratio of variance among subjects to the total variance based on repeated measurements: (BMS-WMS/2)/((BMSWMS/2) +WMS); BMS = between group mean square, WMS = within group mean square (Fleiss JL: Design and Analysis of Clinical Experiments. New York: Wiley-Interscience; 1986:8–13.)


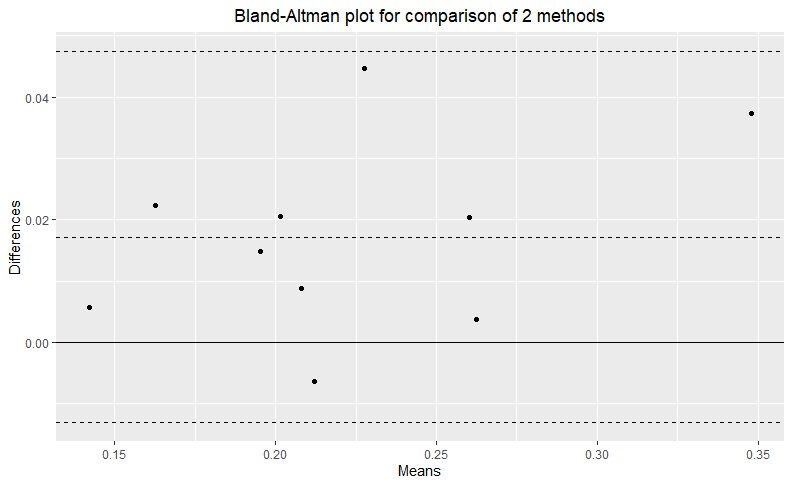

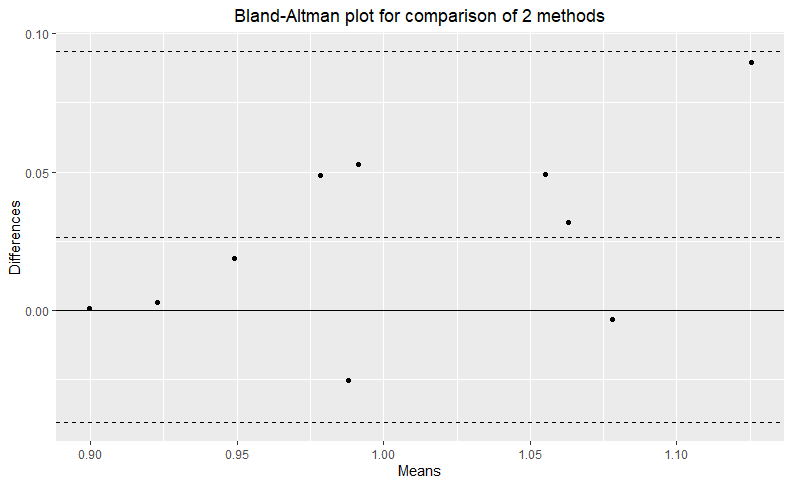

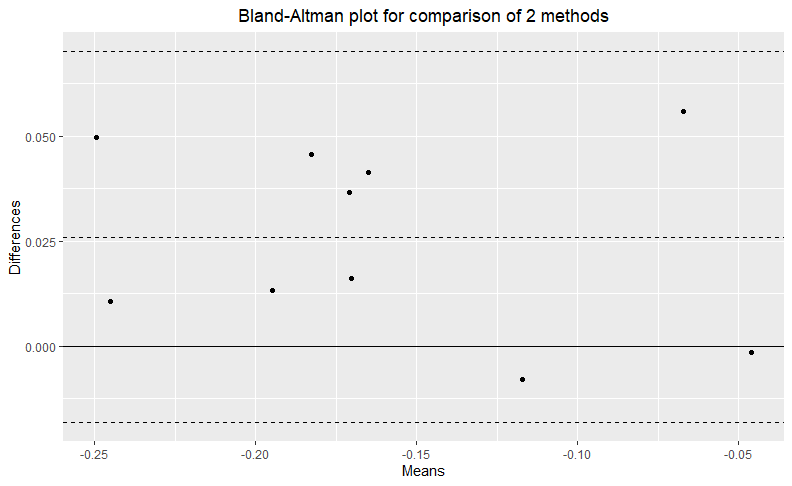

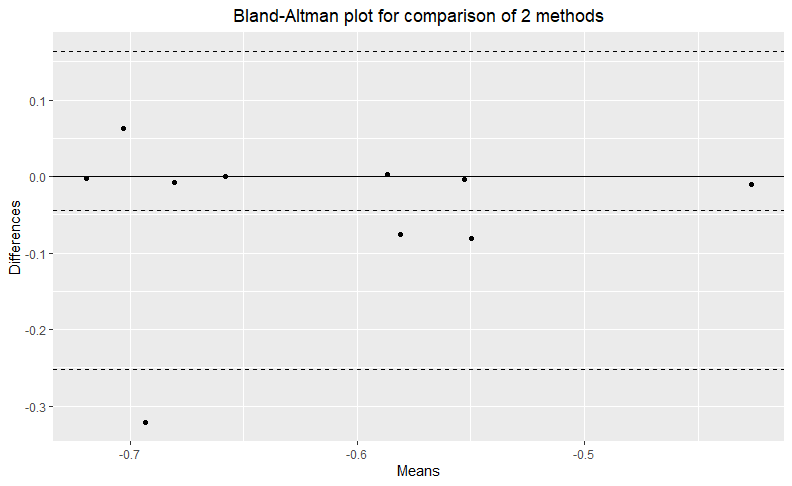


PC(30:0)

PC(33:0)

PC(O-32:1)

PC(36:1)


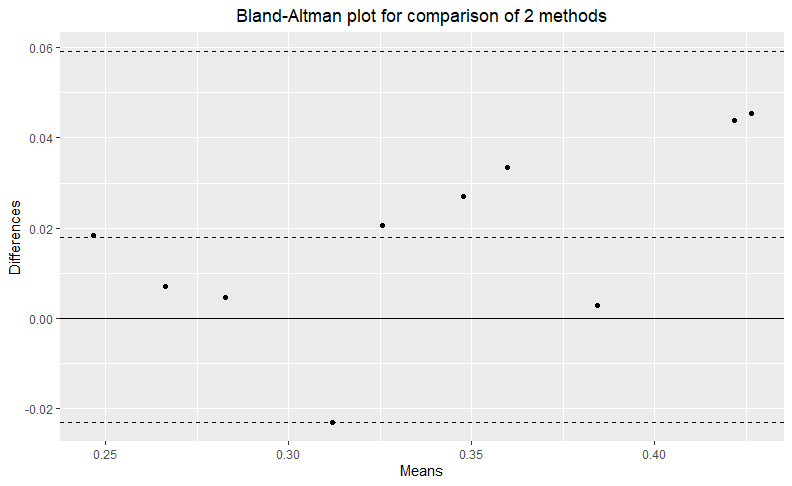

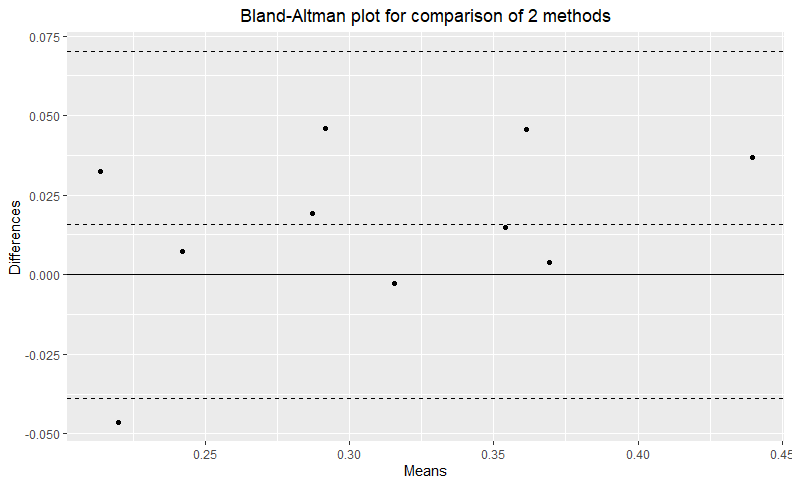

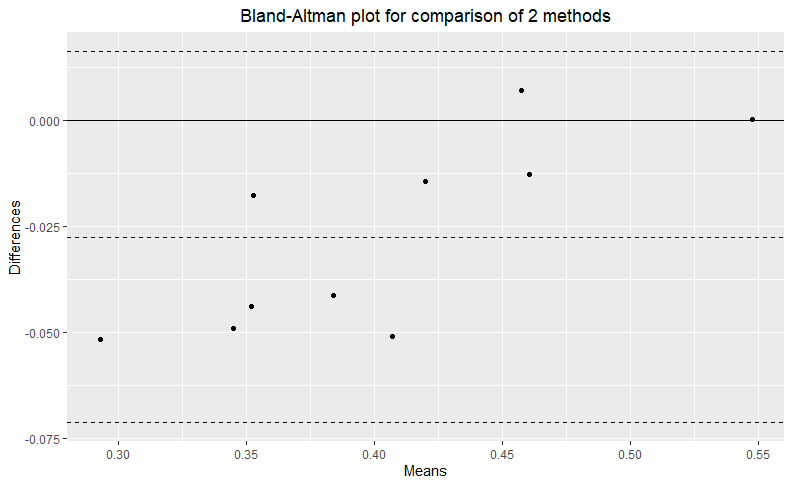

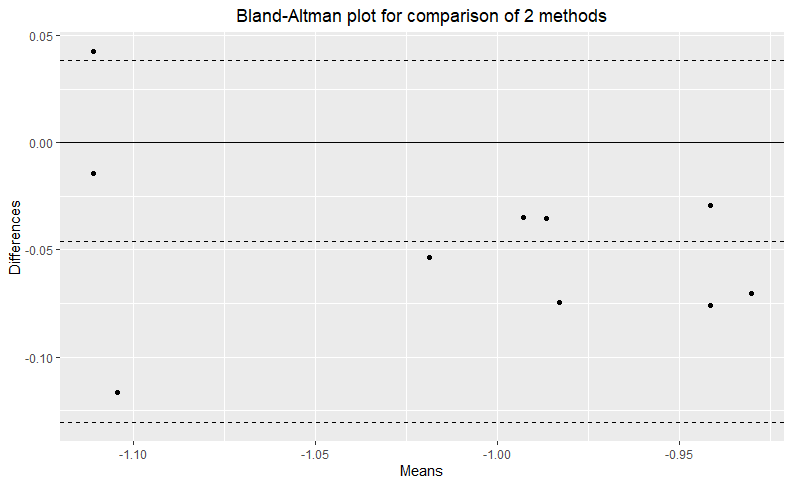


PC(31:0)

PC(34:0)

SM(d34:1)

PC(36:2)


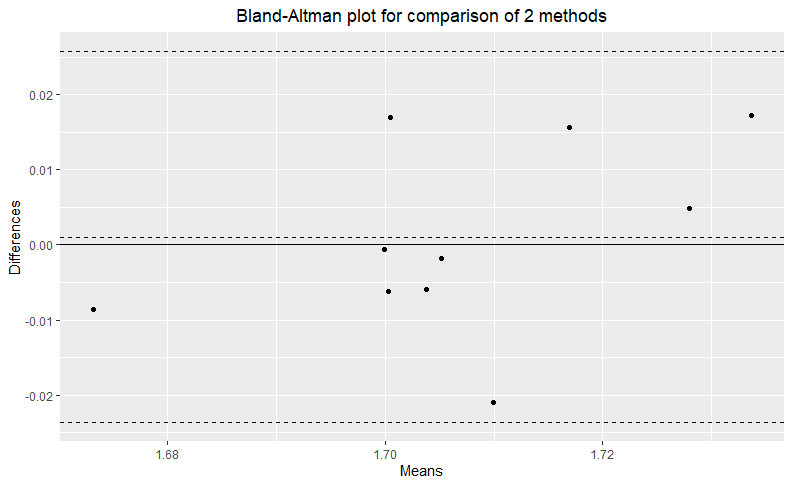

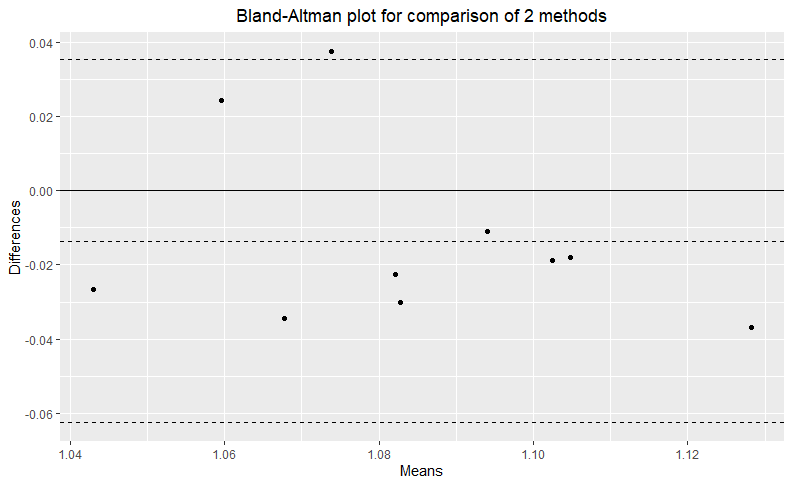

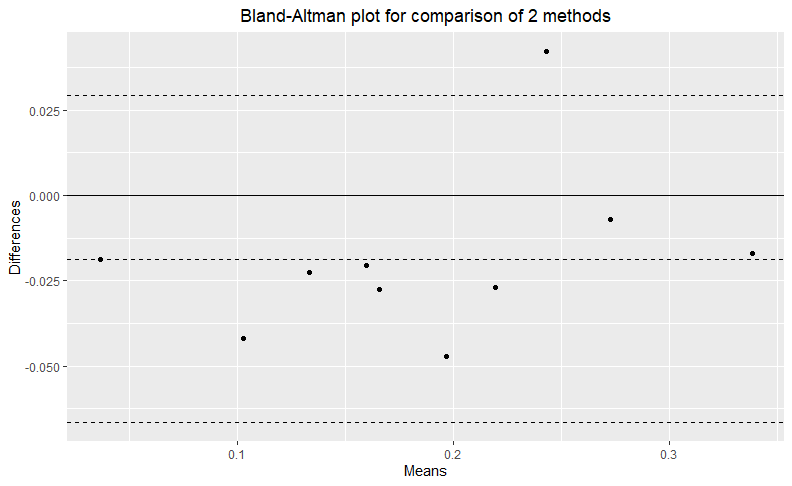

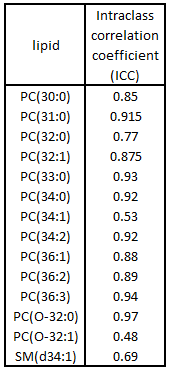


PC(32:0)

PC(36:3)

PC(34:1)


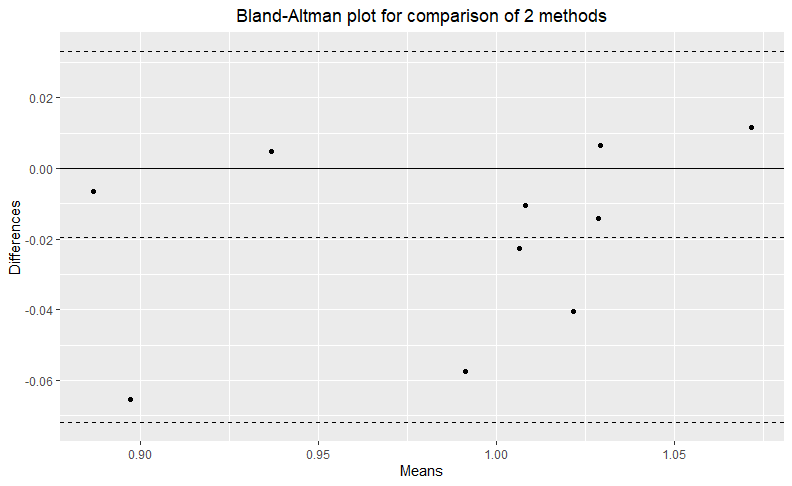

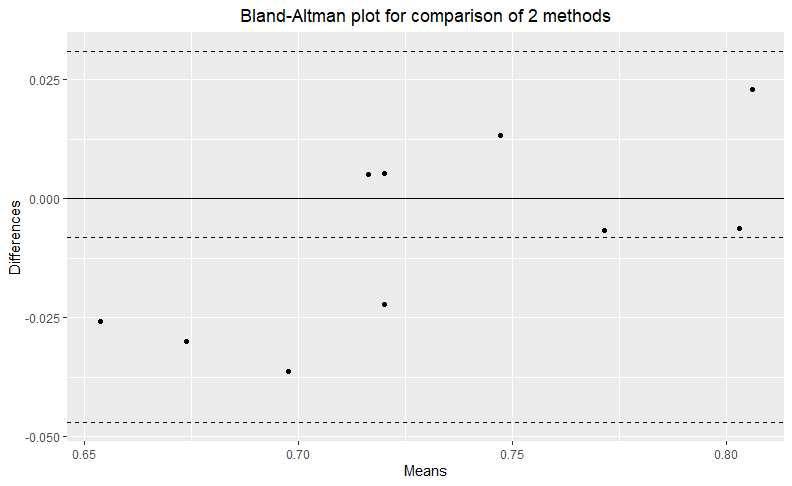

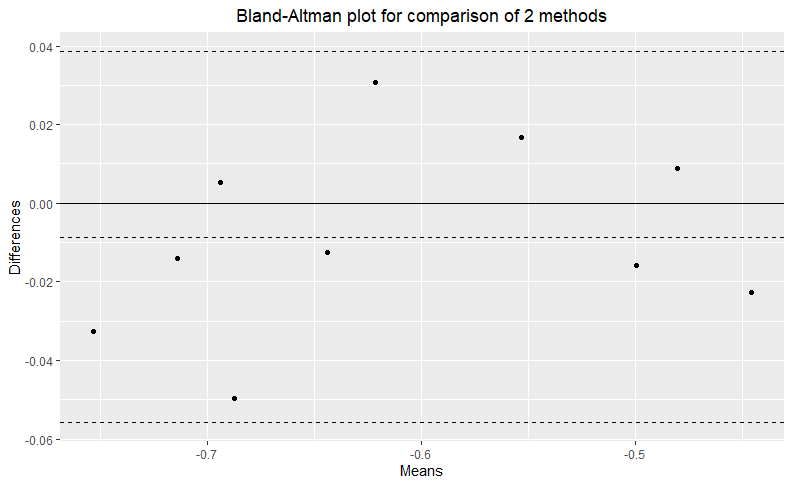


PC(32:1)

PC(34:2)

PC(O-32:0)

**Main manuscript Figure 1 supplementary info: Multivariate models**

**PCA model**

The PCA model is an unsupervised method to summarize the lipid data into two principal components while retaining as much of the variance as possible. Strong groupings can be identified but PCA is not the best method finding variables important for class identification. The main purpose is to examine if there are samples that are strong outliers that would be problematic for OPLS-DA modelling. The lipid variables were scaled to unit variance and centred prior to modelling in all models.

**Figure 1a) PCA model parameters (N=28)**

| **Component** | **R2X** | **R2X(cum)** | **Eigenvalue** | **Q2** | **Q2(cum)** |
| --- | --- | --- | --- | --- | --- |
| 0 | Cent. |  |  |  |  |
| 1 | 0.322 | 0.322 | 4.51 | 0.125 | 0.125 |
| 2 | 0.247 | 0.569 | 3.45 | 0.123 | 0.233 |

**Orthogonal partial least square-discriminant analysis (OPLS-DA)**

OPLS-DA was used for finding the lipids differentiated most strongly between two sampling methods.

The most important parameters for model quality assessment

- **R2Y** Fraction of Y variation modelled in that component, using the X model (R2 = 1.0 explains 100% of the data).
- **Q2** is a cross validation measure of the model’s goodness of fit in the range of 0–1, with the upper limit of 1 for a perfect fit and 0.5 for an acceptable fit. Fraction of Y variation predicted by the X model. In the cross validation for this study the software excludes all data from one individual at a time and recalculates the model iteratively. The predictive quality if the model is estimated by how well all the excluded Y-values were predicted in the models. In an ideal model the R2 and Q2 should be similar, meaning that each of the subjects contribute equally and uniformly to the observed group separation.
- **R2X** **predictive** describes how much of the R2X component that is directly related to the shift between the Y-Var (sampling methods).

Additional model data that may be relevant: **S-plot** is common alternative to the loading plot to illustrate results from OPLS-DA. The S-plots (Figure S3a, S4a, and S5a) provide visualization of the OPLS/OPLS-DA predictive component loading to facilitate model interpretation. **CrossValidation-ANOVA** shows the p-value for the model ability to separate the groups. **Permutation tests** (Figure S3b, S4b, and S5b) is as a measure of how overfitted the models are, permutation of the values should lower Q2 and R2Y.

**Figure 1d) BAL vs PEx (N=19)**

Model was made using one predictive and two orthogonal OPLS components with the resulting model parameters

- R2Y=0.923
- Q2=0.838
- R2X predictive component=0.154

CrossValidation-ANOVA

| M6 | SS | DF | MS | F | p | SD |
| --- | --- | --- | --- | --- | --- | --- |
| Total corr. | 18 | 18 | 1 |  |  | 1 |
| Regression | 15.09 | 6 | 2.52 | 10.38 | **3.70E-04** | 1.59 |
| Residual | 2.91 | 12 | 0.24 |  |  | 0.49 |
|  |  |  |  |  |  |  |

**Figure S3a: Figure S3b:**

S-Plot: Showing model reliability and model influence for the lipids. Permutation Plot: test for overfitting


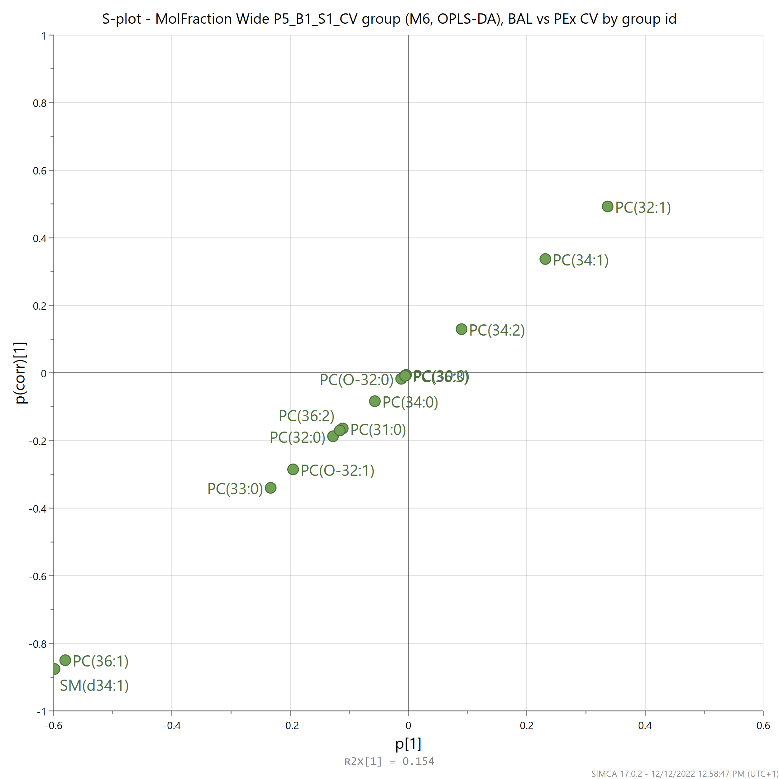

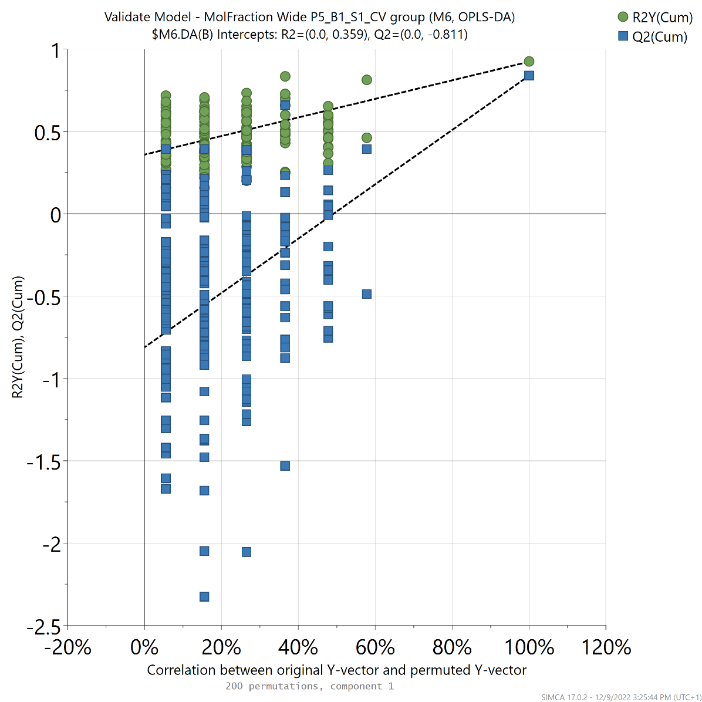


**Figure 1e) BAL vs ISP (N=19)**

Model was made using one predictive and one orthogonal OPLS components with the resulting model parameters

- R2Y=0.867
- Q2=0.805
- R2X predictive component =0.274

CrossValidation-ANOVA

| M7 | SS | DF | MS | F | p | SD |
| --- | --- | --- | --- | --- | --- | --- |
| Total corr. | 18 | 18 | 1 |  |  | 1 |
| Regression | 14.49 | 4 | 3.62 | 14.4423 | **7.13E-05** | 1.90 |
| Residual | 3.51 | 14 | 0.25 |  |  | 0.50 |

**Figure S4a: Figure S4b:**

S-Plot: Showing model reliability and model influence for the lipids Permutation Plot: test for overfitting


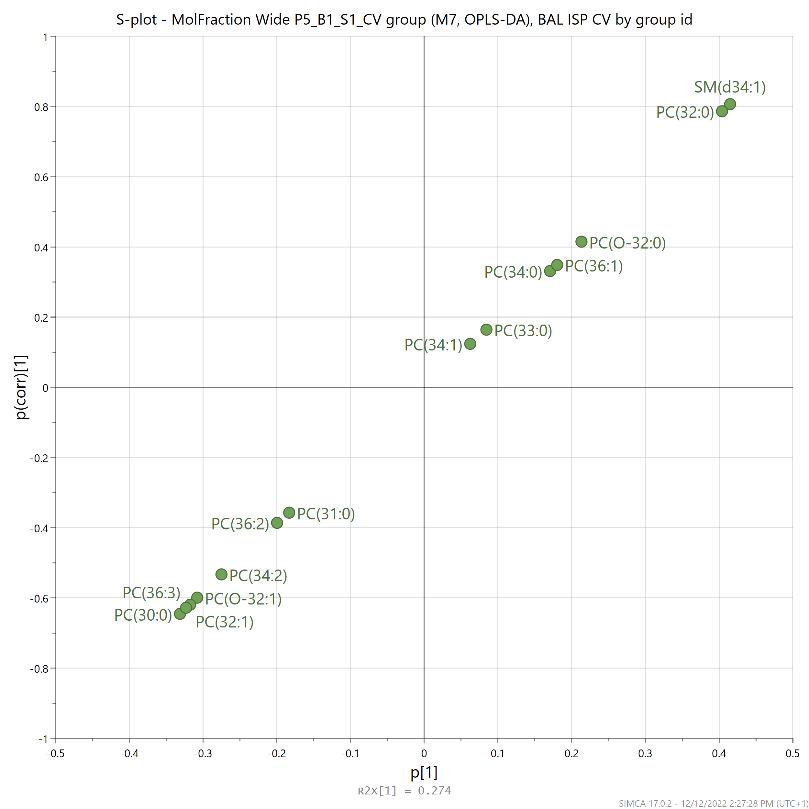

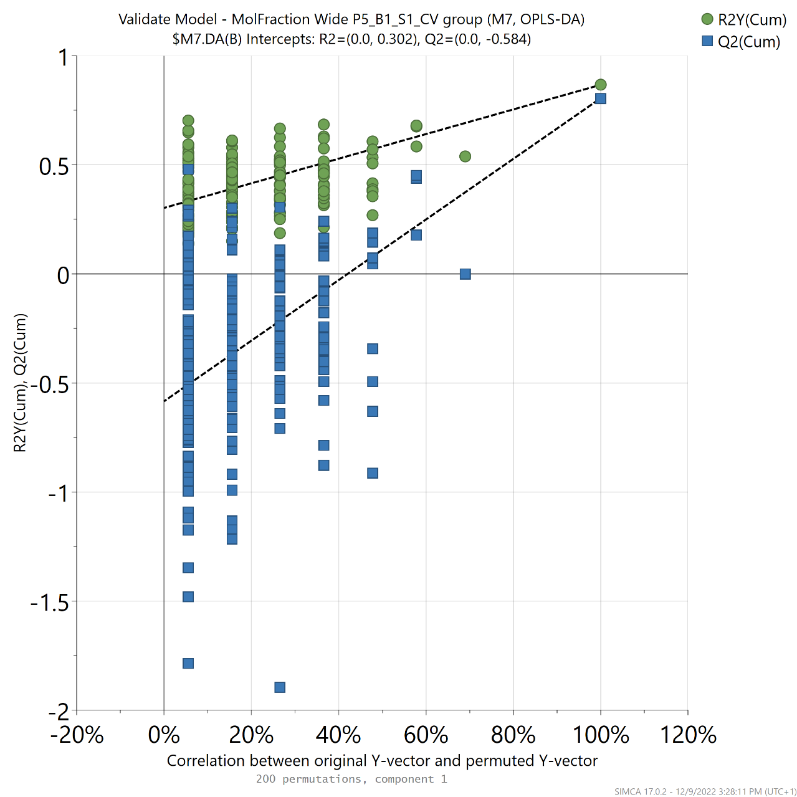


**Figure 1f) PEx vs ISP (N=18)**

Model was made using one predictive and no orthogonal OPLS components with the resulting model parameters.

- R2Y=0.900
- Q2=0.894
- R2X predictive component =0.390

CrossValidation-ANOVA

| M5 | SS | DF | MS | F | p | SD |
| --- | --- | --- | --- | --- | --- | --- |
| Total corr. | 17 | 17 | 1 |  |  | 1 |
| Regression | 15.20 | 2 | 7.60 | 63.39 | 4.83E-08 | 2.76 |
| Residual | 1.80 | 15 | 0.12 |  |  | 0.35 |

**Figure S5a: Figure S5b:**

S-Plot: Showing model reliability and model influence for the lipids. Permutation Plot: test for overfitting
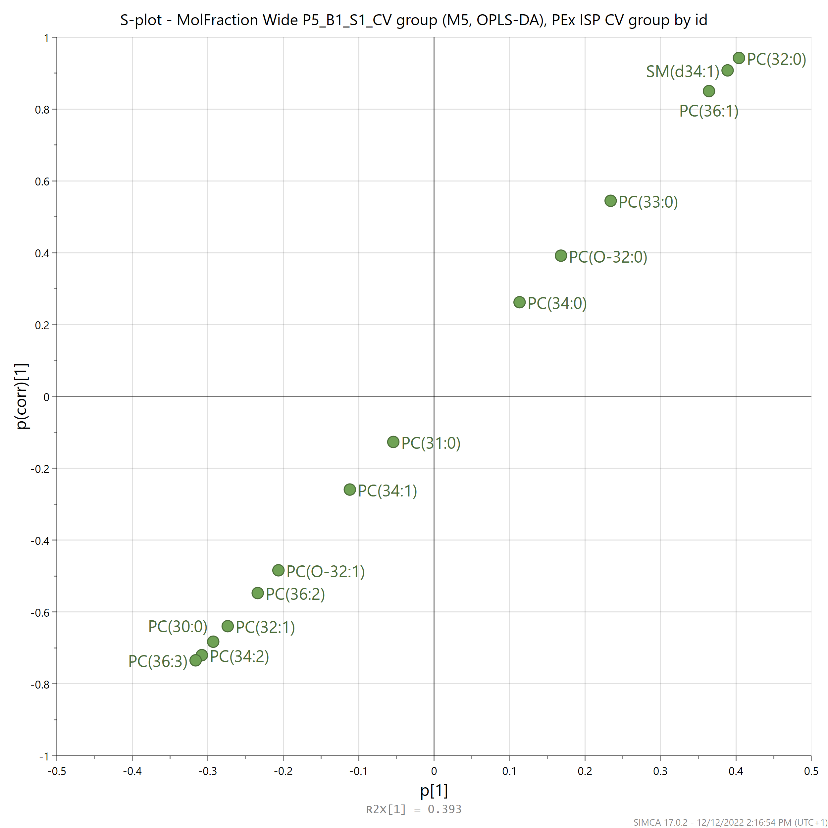

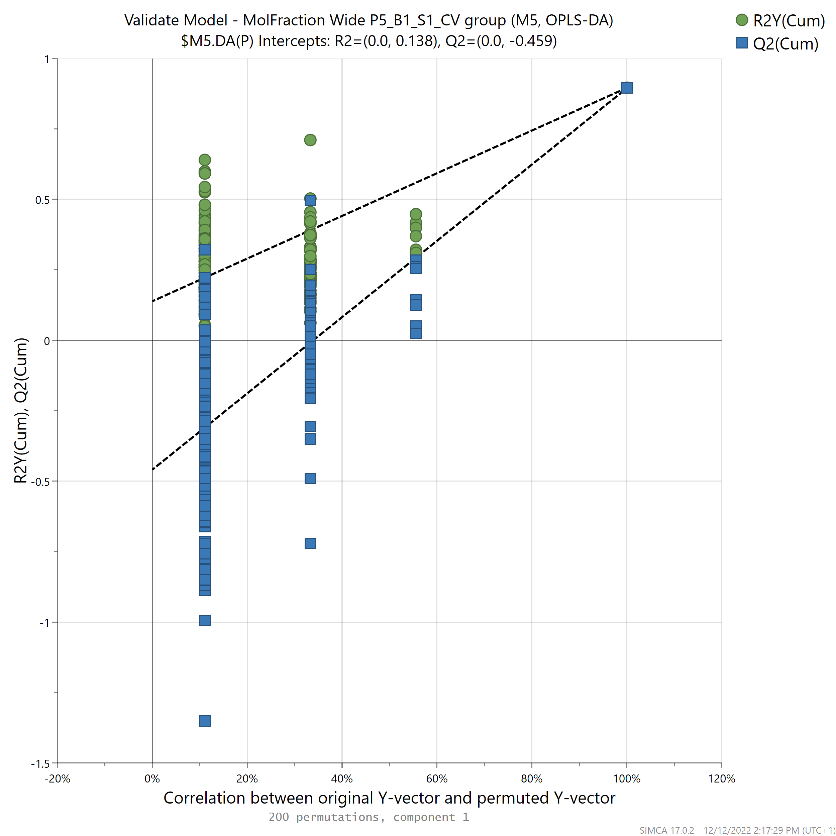


**Raw data from LC-MSMS analysis with shown chromatograms**

The response factor is the ratio of the analyte to the spiked internal standard area. Each sample was analyzed using two injection and the RSD% was on average 2.4 (range 0-14%). SMd34:1 in PEx sample from id 7715 had the highest RSD% (RSD%=13.4) and among the lowest signals. For this sample, chromatograms for lowest signal are shown below (Figure S6a-d). The signal for the blank extracted sample substrate is also shown to illustrate how a low PEx sample compare to a sample without any collected PEx material. Each print screen has two chromatograms, the upper is the analyte signal and the lower is the internal standard signal.

**Figure S6: Chromatogram for PEx sample for ID7715 and analyte SMd34:1**

**Figure S6a: ID7715 _PEx_sample5_A_injection lipid SMd34:1**


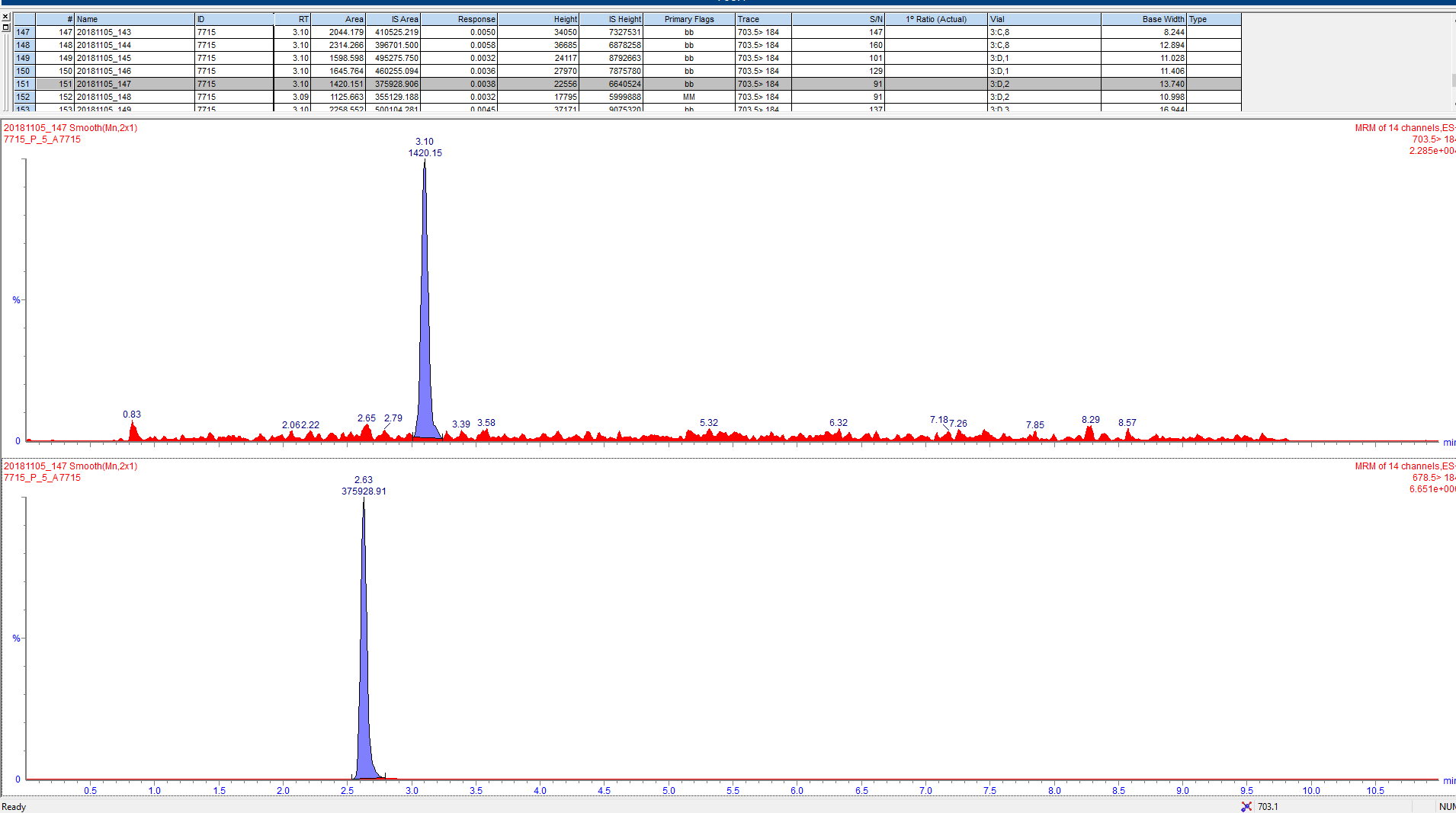


**Figure S6b: ID7715 _PEx_sample5_B_injection lipid SMd34:1**


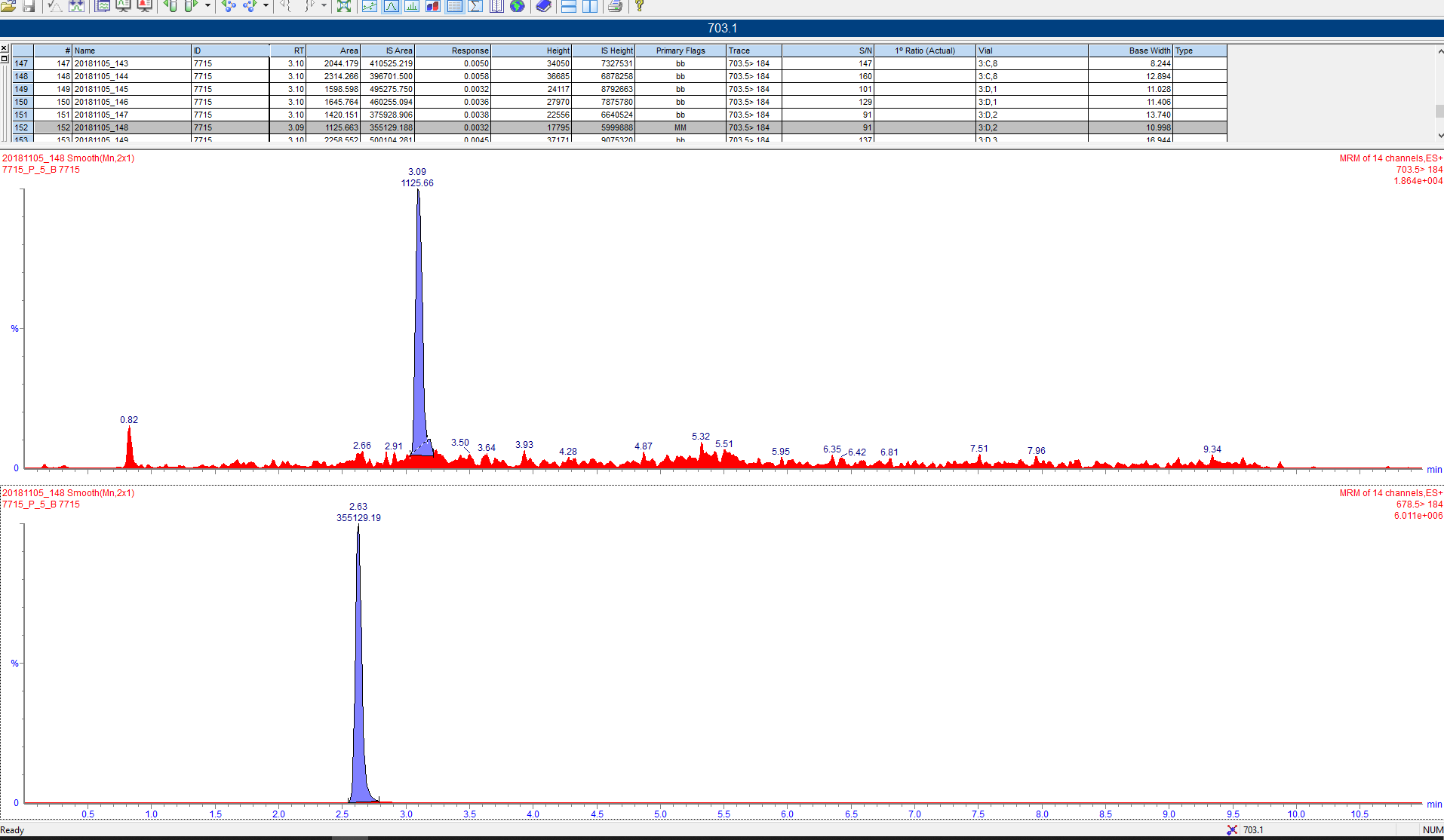


**Figure S6c: Blank sample substrate IS spiked SMd34:1 background signal extraction and analysis injection A**


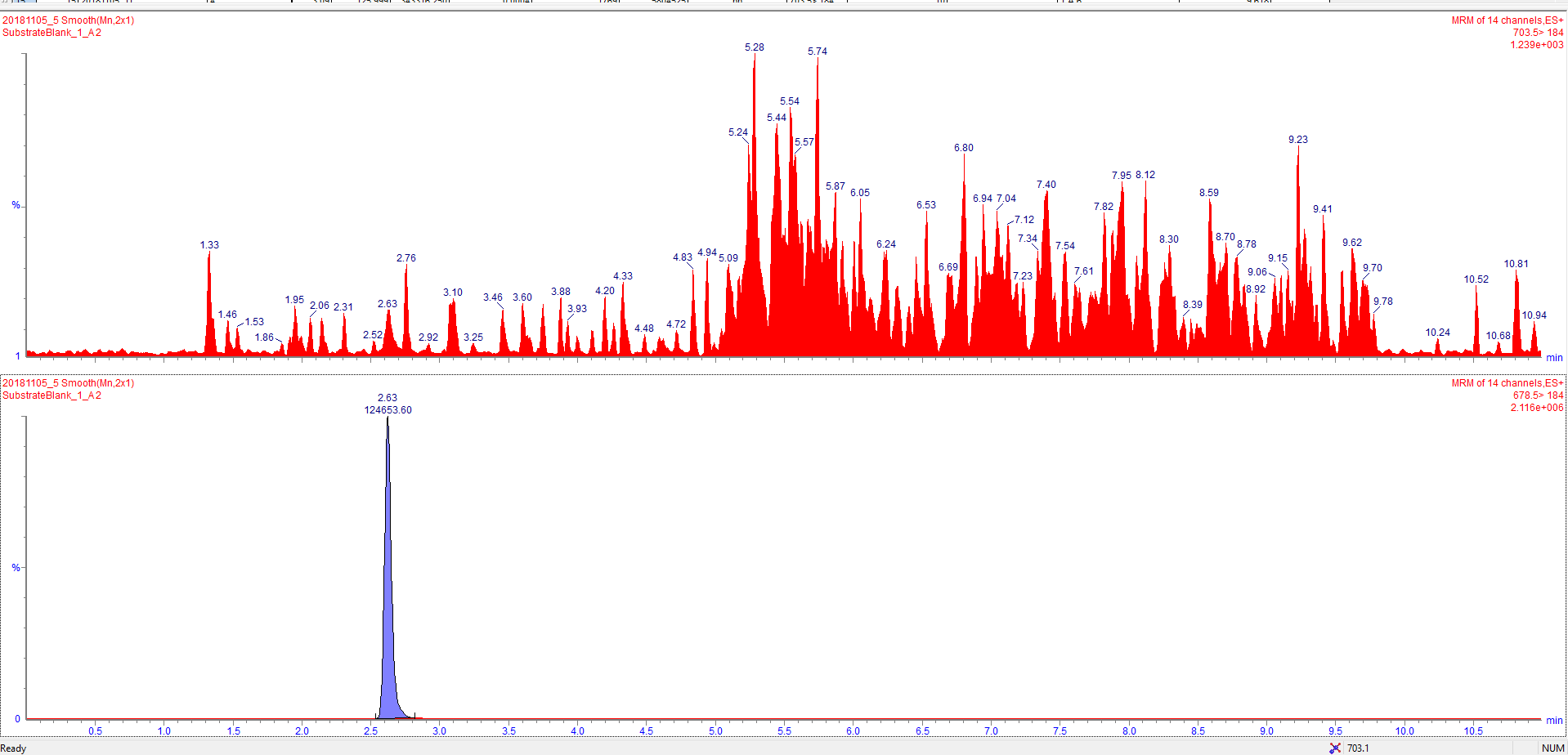


**Figure S6d: Blank sample substrate IS spiked SMd34:1 background signal extraction and analysis injection B**


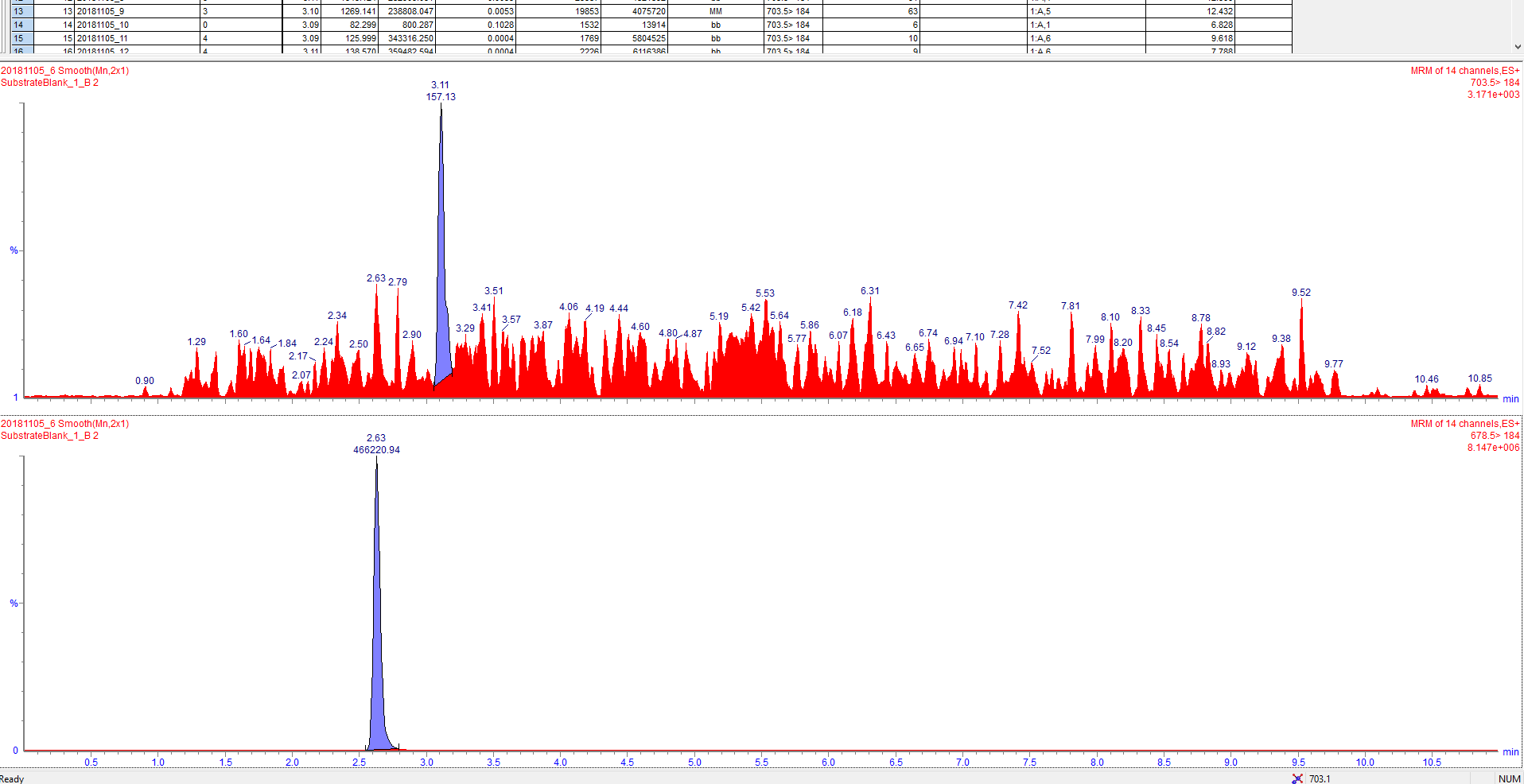


Summary table of mixed model results (sampling method as the fixed effect and the individual as the random effect)
